# A combination of curcumin and oligomeric proanthocyanidins offer superior anti-tumorigenic properties in colorectal cancer

**DOI:** 10.1038/s41598-018-32267-8

**Published:** 2018-09-14

**Authors:** Preethi Ravindranathan, Divya Pasham, Uthra Balaji, Jacob Cardenas, Jinghua Gu, Shusuke Toden, Ajay Goel

**Affiliations:** 1 Center for Gastrointestinal Research, Center for Translational Genomics and Oncology, Baylor Scott & White Research Institute and Charles A Sammons Cancer Center, Baylor University Medical Center, Dallas, Texas USA; 2 Baylor Scott & White Research Institute, Baylor University Medical Center, Dallas, Texas USA

## Abstract

Combining anti-cancer agents in cancer therapies is becoming increasingly popular due to improved efficacy, reduced toxicity and decreased emergence of resistance. Here, we test the hypothesis that dietary agents such as oligomeric proanthocyanidins (OPCs) and curcumin cooperatively modulate cancer-associated cellular mechanisms to inhibit carcinogenesis. By a series of *in vitro* assays in colorectal cancer cell lines, we showed that the anti-tumorigenic properties of the OPCs-curcumin combination were superior to the effects of individual compounds. By RNA-sequencing based gene-expression profiling in six colorectal cancer cell lines, we identified the cooperative modulation of key cancer-associated pathways such as DNA replication and cell cycle pathways. Moreover, several pathways, including protein export, glutathione metabolism and porphyrin metabolism were more effectively modulated by the combination of OPCs and curcumin. We validated genes belonging to these pathways, such as HSPA5, SEC61B, G6PD, HMOX1 and PDE3B to be cooperatively modulated by the OPCs-curcumin combination. We further confirmed that the OPCs-curcumin combination more potently suppresses colorectal carcinogenesis and modulated expression of genes identified by RNA-sequencing in mice xenografts and in colorectal cancer patient-derived organoids. Overall, by delineating the cooperative mechanisms of action of OPCs and curcumin, we make a case for the clinical co-administration of curcumin and OPCs as a treatment therapy for patients with colorectal cancer.

## Introduction

**Figure 1:**
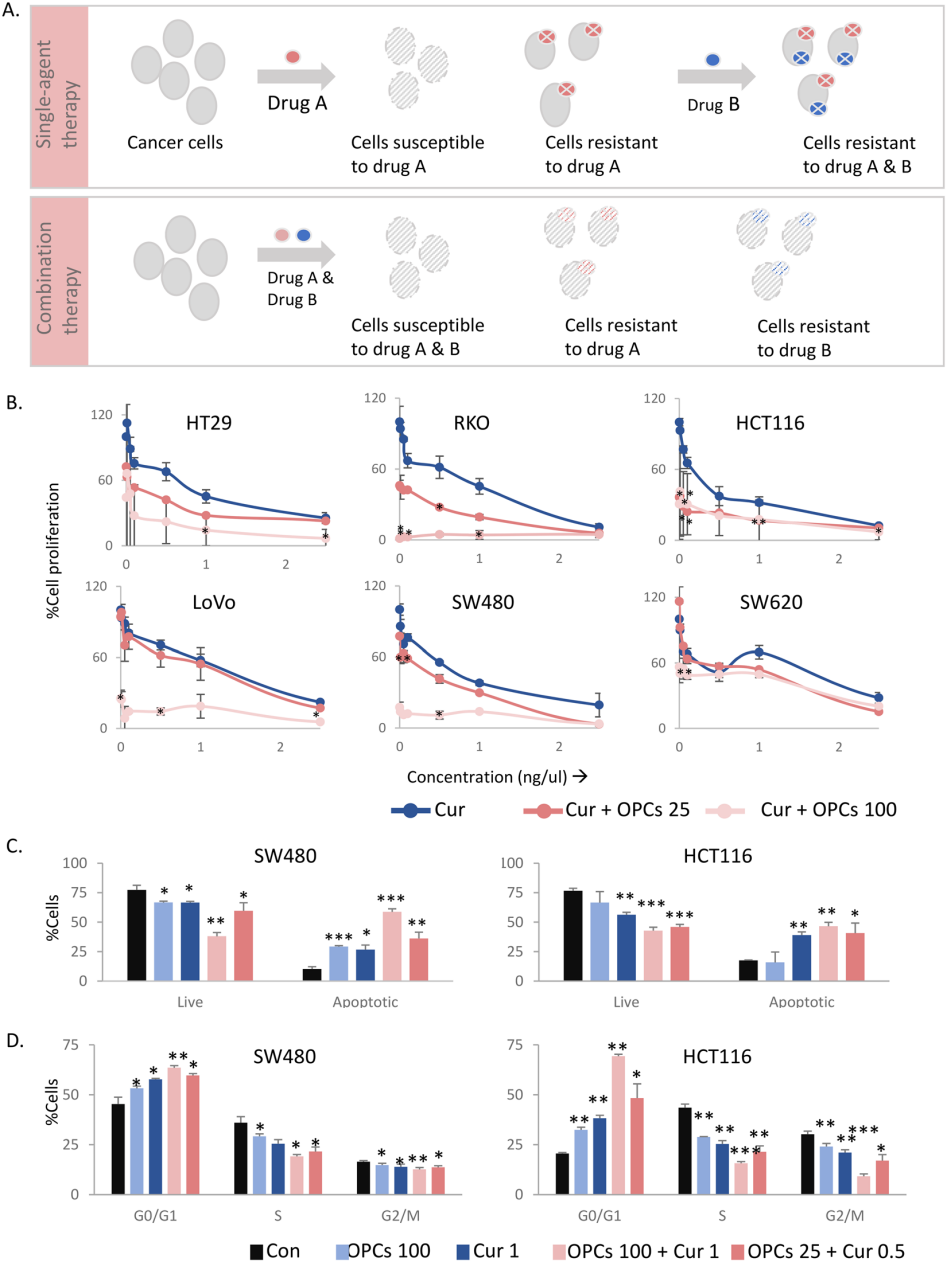
The combination of curcumin and OPCs has superior anti-cancer effects than individually. (**A**) Top: Schematic illustration of the development of chemo-resistance against drug(s) used as the sole agent or in sequence with another agent. Bottom: Schematic showing the rational for combining drugs with different mechanisms of anti-cancer action in cancer therapy. (**B**) Cell proliferation assay showing the effect of curcumin alone or in combination with OPCs at different doses in six CRC cells. X-axis shows the doses of curcumin in ng/ul. Y-axis shows the percentage change in cell count with respect to control cells treated with curcumin alone at the corresponding dose. (**C**) Percentage of cells undergoing apoptosis as measured by percentage that stained positive for Annexin-V assay (**D**) Analysis of effect on cell cycle as evaluated by the DNA staining of propidium iodide in HCT116 and SW480 cells treated with indicated concentrations of OPCs or curcumin. *P < 0.05, **P < 0.01, ***P < 0.001 compared to control treatments.

The development of chemoresistance presents a major challenge in the treatment and management of colorectal cancer (CRC; Fig. 1A). When treated with a chemotherapeutic agent that targets a specific mechanism, the tumor relapses due to compensatory mutations or activation of alternative signaling pathways in subsets of cancer cells^1,2^. Switching to a second agent after the tumor relapses is usually ineffective due to ensuing compensatory mechanisms against the second agent^3^. Alternatively, combining agents that coordinately modulate multiple tumorigenic mechanisms often demonstrate enhanced therapeutic efficacy due to the lower likelihood of resistance emerging against both agents in the cancer cells. Moreover, the dosage of individual drugs is often smaller in drug combinations, diminishing the toxic effects caused by higher doses of a single therapeutic agent^4,5^.

Emerging evidence indicates that several dietary agents possess anti-tumorigenic properties by targeting multiple oncogenic signaling pathways^6–11^. In particular, two dietary agents, curcumin^12–14^ and oligomeric proanthocyanidins (OPCs)^15^, have been extensively studied for their anti-cancer properties in CRC. Curcumin, a dietary polyphenol derived from the spice turmeric, has been shown to exert chemopreventive and anti-tumor effects by modulating transcription factors and signaling pathways, including PI3K/mTOR, Ras/Raf/MEK and GSK-3beta pathways^16–21^. In addition, we recently delineated the anti-cancer mechanisms of OPCs, a group of flavonoids from grape seeds, in CRC by genome-wide mRNA expression profiling (unpublished results). Given their diverse anti-cancer mechanisms, we hypothesized that co-administering OPCs and curcumin might cooperatively modulate multiple cancer-associated signaling pathways and, thereby be more effective against CRC. Furthermore, a combination of OPCs and curcumin at minimum tolerable doses might effectively decrease tumorigenesis with minimal concurring toxicity and lower the prospect of acquiring drug resistance.

Adjunctive treatment with curcumin together with conventional cytotoxic drugs including 5-fluorouracil, cisplatin and mitomycin, have been shown to improve the overall chemotherapeutic response^22–25^. However, the underlying mechanisms by which curcumin enhances efficacy of these compounds remain unclear. Herein, through a series of systematic cell lines, animal model and patient-derived tumor organoid experiments, we for the first time report that the combination of curcumin and OPCs has a superior anti-tumorigenic effect in colorectal cancer. Subsequently, we sought to identify the underlying mechanism(s) by which the two agents cooperatively hinder colorectal tumor progression. Although a comprehensive analysis of all the genes regulated by curcumin has been investigated in a variety of cancers, including melanoma^26^, breast^27^ and lung^28^ cancers, such an assessment in CRC has not been accomplished to date. Therefore, we performed genome-wide RNA-sequencing in a panel of CRC cell lines treated with curcumin and/or OPCs, and interrogated signaling pathways modulated by these two compounds. By comparing the genes regulated by OPCs in CRC cell lines, we discovered that though curcumin and OPCs cooperatively affect several critical mechanisms of carcinogenesis, each treatment also affects distinctive signaling pathways, making them a promising combination in cancer therapy.

Overall, this study reveals that curcumin and OPCs potently inhibit colorectal carcinogenesis by cooperatively modulating multiple cancer-associated mechanisms. The anti-tumor response to the combination of curcumin and OPCs in mice xenograft tumors and organoids derived from primary human CRC tumors correlated with the altered gene expressions of HSPA5, IHH, PDE3B, cyclin D1 and SEC61B. Collectively, our data presents the possibility of combining curcumin and OPCs to curb colorectal carcinogenesis, which has the potential for leveraging as a safe and inexpensive therapeutic option, on its own or in combination with conventional chemotherapeutic drugs.

## Results

### Curcumin and OPCs cooperatively inhibit cellular growth in colorectal cancer cells

The anti-tumorigenic properties of OPCs and curcumin in CRC are well-established^15,29–36^. In order to study the effect of the combination of curcumin and OPCs, we used six colorectal cancer cell lines that have been well-studies and broadly represent the common mutational and microsatellite statuses found in colorectal cancer. We found that the combination was more effective in inhibiting cell proliferation compared to the individual agents (Fig. 1B). To determine any evidence of the cooperativity between OPCs and curcumin, we calculated the Combination Index (CI) for OPCs-curcumin combinations by two methods. The first method, called the ‘Highest Single Agent’ method (Supplemental Fig. 1, shown for OPCs at 25 ng/ul and curcumin at 0.5 ng/ul), measures if the resulting effect of the combination is greater than the individual agents. Our second method utilized the ‘Bliss Independence’ model (Supplemental Fig. 2, shown for OPCs at 25 ng/ul and curcumin at 0.5 ng/ul), which is the multiplicative probability assuming the agents act independently toward a common result. The Combination Indices (CI) calculated by both methods were less than 1 for most cell lines, suggesting strong cooperative action between curcumin and OPCs.

Additionally, to measure the extent to which the doses of curcumin or OPCs can be reduced in the combination to derive comparable efficacy as the individual agents, we calculated the ‘Dose Reduction Index (DRI)’ (Supplemental Table 3). The values of the DRI were above 1 for most cell lines, highlighting a beneficial combination.

We then assessed whether the reduced cellular growth observed in the cell proliferation assay by OPCs-curcumin combination was due to apoptosis using a flow cytometry-based assay in two representative cell lines: MSI cell line HCT116 and MSS cell line SW480. Both OPCs (100 ng/ul) and curcumin (1 ng/ul) induced apoptosis in HCT116 and SW480 cells, and their combination at full doses (OPCs at 100 ng/ul and curcumin at 1 ng/ul) further significantly enhanced apoptosis rates in these cells (p = 0.0002 for SW480 cells, p = 0.0014 for HCT116 cells; Fig. 1C). Interestingly, combining OPCs at a much lower dose of 25 ng/ul with curcumin at 0.5 ng/ul still induced apoptosis in HCT116 and SW480 cells at levels comparable to those of the full doses of OPCs and curcumin, supporting the rationale for combined treatment with these two botanicals.

Additionally, we found that while both OPCs (100 ng/ul) and curcumin (0.5 ng/ul) individually caused cell cycle arrest in HCT116 (p = 0.0016 for OPCs, 0.0016 for curcumin) and SW480 (p = 0.028 for OPCs, 0.02 for curcumin) cells, a combination of low doses of OPCs at only 25 ng/ul and curcumin of 0.5 ng/ul induced a comparable and significant cell cycle arrest (p = 0.01 for SW480 cells; p = 0.02 HCT116 cells) (Fig. 1D). Taken together, these data suggest the superiority of the anti-cancer properties of the combination of OPCs and curcumin over the individual agents.

### Curcumin and OPCs modulate multiple cancer-associated pathways

**Figure 2:**
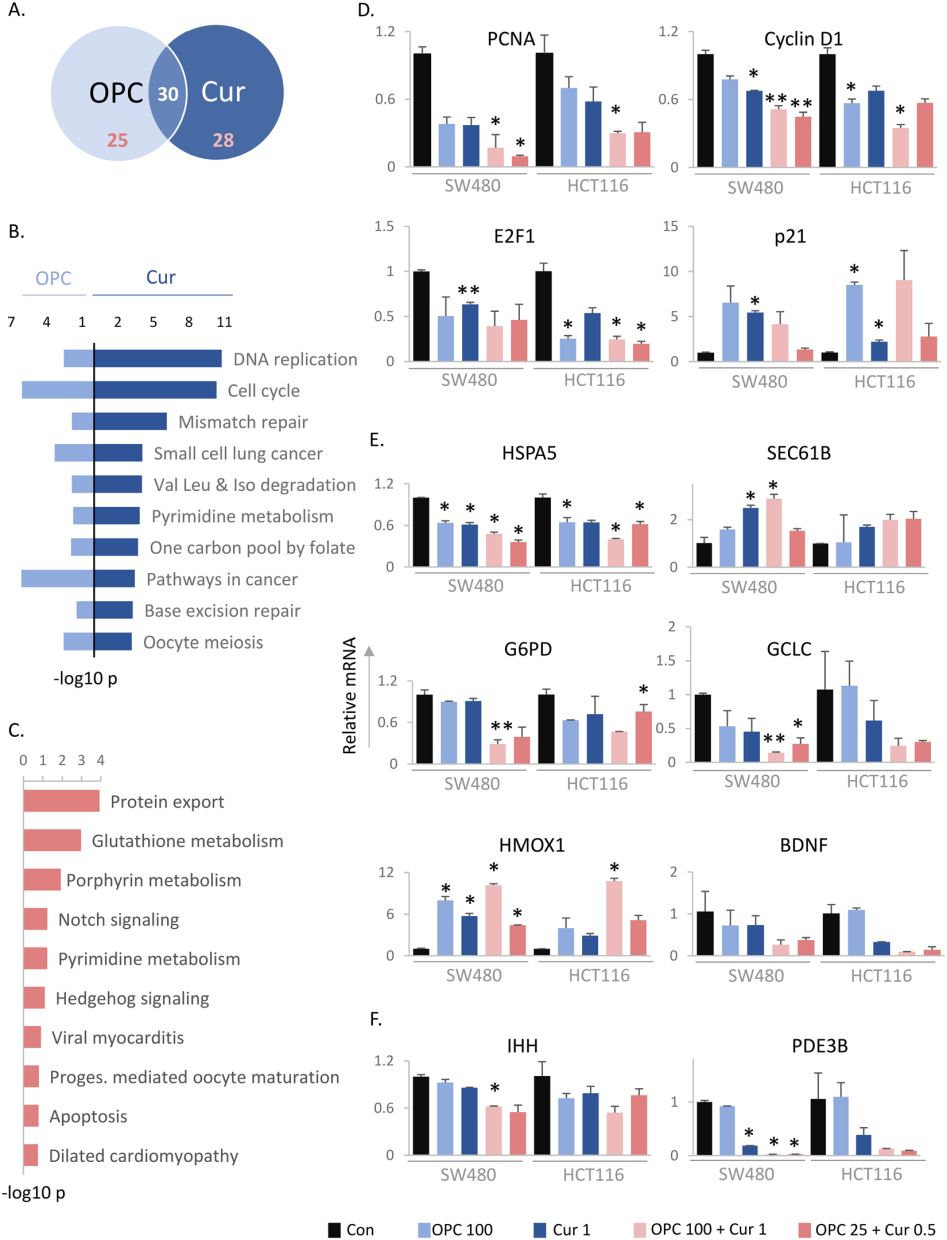
OPCs and curcumin affect several molecular pathways. (**A**) Venn diagram showing the number of common and unique KEGG pathways affected by curcumin or OPCs in six CRC cell lines. (**B**) The top 10 pathways affected by both OPCs and curcumin. (**C**) Top pathways cooperatively regulated by OPCs and curcumin. (**D**) Levels of mRNA of genes belonging to pathways commonly affected by both OPCs and curcumin, normalized to mRNA levels of β-actin. (**E**) Levels of mRNA of genes belonging to top pathways cooperatively affected by OPCs and curcumin, namely (top) HSPA5 and SEC. 61 from protein export pathway, (middle) G6PD and GCLC from glutathione metabolism, and (bottom) HMOX1 and BDNF from porphyrin metabolism pathways, normalized to mRNA levels of β-actin. (**F**) mRNA levels of genes from pathways activated only by OPCs-curcumin combination and not by the individual agents, normalized to mRNA levels of β-actin. *P < 0.05, **P < 0.01, ***P < 0.001 compared to control treatments.

Encouraged by the results of our *in vitro* assays, we wanted to examine the underlying molecular mechanisms by which OPCs and curcumin cooperatively function against CRC. Thus, we looked at the genome-wide changes in gene expression induced by either OPCs or curcumin and their combination in six CRC cell lines SW480, SW620, HT29, HCT116, RKO and LoVo using RNA sequencing. KEGG pathway analysis of the differentially expressed genes relative to untreated cells revealed 30 pathways that were commonly regulated by both OPCs and curcumin, 25 pathways that were uniquely regulated by OPCs and 28 pathways uniquely regulated by curcumin (Fig. 2A). Consistent with our previously unpublished data on OPCs, the top pathways that were commonly regulated by both OPCs and curcumin were DNA replication (p = 1.7e-11 for curcumin, p = 0.003 for OPCs) and cell cycle (p = 4.7e-11 for curcumin, p = 8.2e-07 for OPCs; Fig. 2B). We validated the RNA-sequencing data experimentally by measuring changes in mRNA levels of well-recognized key genes associated with DNA replication, PCNA and CCND1 (cyclin D1), and the cell cycle, namely E2F1 and CDKN1a (P21) in HCT116 and SW480 cells (Fig. 2D). While OPCs and curcumin both decreased the mRNA levels of proliferation markers PCNA and cyclin D1, the OPCs-curcumin combination further decreased their mRNA levels in both HCT116 and SW480 cells (Fig. 2D, top). Additionally, the combination of OPCs and curcumin at lower doses of 25 ng/ul and 0.5 ng/ul respectively, were as effective as the individual doses in decreasing the levels of PCNA and cyclin D1. The expression of certain genes, such as the transcription factor E2F1, was decreased by both OPCs and curcumin individually, and the OPCs-curcumin combination did not further decrease their expression (Fig. 2D, bottom left). Other genes, such as p21, were better regulated by OPCs than curcumin (Fig. 2D, bottom right).

Moreover, based on the pathway analysis, steroid biosynthesis (p = 8.8e-05) and axon guidance (p = 0.3 e-04) were among the top pathways regulated exclusively by OPCs (Supplemental Fig. 3A). The top pathways regulated exclusively by curcumin were glutathione metabolism (p = 1.2e-06) and spliceosome (p = 6.8e-06) pathways. Taken together, these data illustrate the diverse mechanisms by which OPCs and curcumin impede carcinogenesis.

### The OPCs-curcumin combination cooperatively modulates cancer-associated pathways

Next, we were curious to identify the pathways that were cooperatively modulated by the combination of OPCs and curcumin. To do this, we identified all the genes differentially altered by the OPCs-curcumin combination and compared to those altered by the compounds individually in at least three cell lines by RNA-sequencing. By KEGG pathway analysis, we identified protein export (p = 1e-04), glutathione metabolism (p = 0.001) and porphyrin-chlorophyll metabolism (p = 0.012) as the top pathways that were altered more by the combination of curcumin and OPCs than by these compounds alone (Fig. 2C).

We next validated alterations in these pathways experimentally by measuring the mRNA levels of key representative genes belonging to these pathways. The expression level of HSPA5, which forms the binding immunoglobulin portion of the chaperone protein Hsp70, and plays a critical role in protein folding and translocation machinery^37^, was decreased by both curcumin and OPCs in HCT116 and SW480 cells, and was further down-regulated significantly when treated with a combination of both compounds (p = 0.025 for SW480 cells, p = 0.049 for HCT116 cells; Fig. 2E, top left). Similarly, the mRNA level of Sec61b, a protein involved in modulating sensitivity to platinum-based chemotherapeutic agents such as oxaliplatin and cisplatin^38^, showed a greater increase with the combination of OPCs and curcumin, vis-à-vis, individual agents (Fig. 2E, top right). Interestingly, while the expression levels of G6PD, a key enzyme in the pentose phosphate pathway and linked to colon carcinogenesis^39,40^, were not altered by curcumin or OPCs alone: the combination of the two compounds significantly decreased its expression (p = 0.0054 for SW480 cells, p = 0.039 for HCT116 cells; Fig. 2E, middle). Likewise, the expression of GCLC, a gene notorious for driving chemoresistance in several cancers^41–43^, was only significantly downregulated by a combination of curcumin and OPCs (p = 0.005 for SW480 cells), not individually (Fig. 2E, middle). We then validated genes from the porphyrin and chlorophyll metabolism pathway, namely HMOX1 and BDNF, both of which were better modulated by the combination of agents than individually (Fig. 2E, lower).

Furthermore, by comparing the KEGG pathways modulated by the OPCs and curcumin combination vs. those modulated by OPCs or curcumin alone, we identified that several molecular pathways, such as hedgehog signaling, PPAR signaling and insulin signaling pathways were uniquely regulated by the combination of OPCs and curcumin, but not by the individual agents (Supplemental Fig. 2B). We confirmed modulation of these pathways by evaluating the levels of IHH and PDE3B by RT-qPCR (Fig. 2F). Curcumin and OPCs in combination suppressed the expression of IHH in both SW480 and HCT116 cells, whereas individual doses of these compounds did not. While the expression of PDE3B was suppressed only by curcumin and not by OPCs, the combination of OPCs and curcumin completely abolished the expression of PDE3B. These results confirm the cooperative modulation of molecular mechanisms by OPCs and curcumin.

### Combination of curcumin and OPCs more effectively decreases tumor growth in mice xenografts

**Figure 3:**
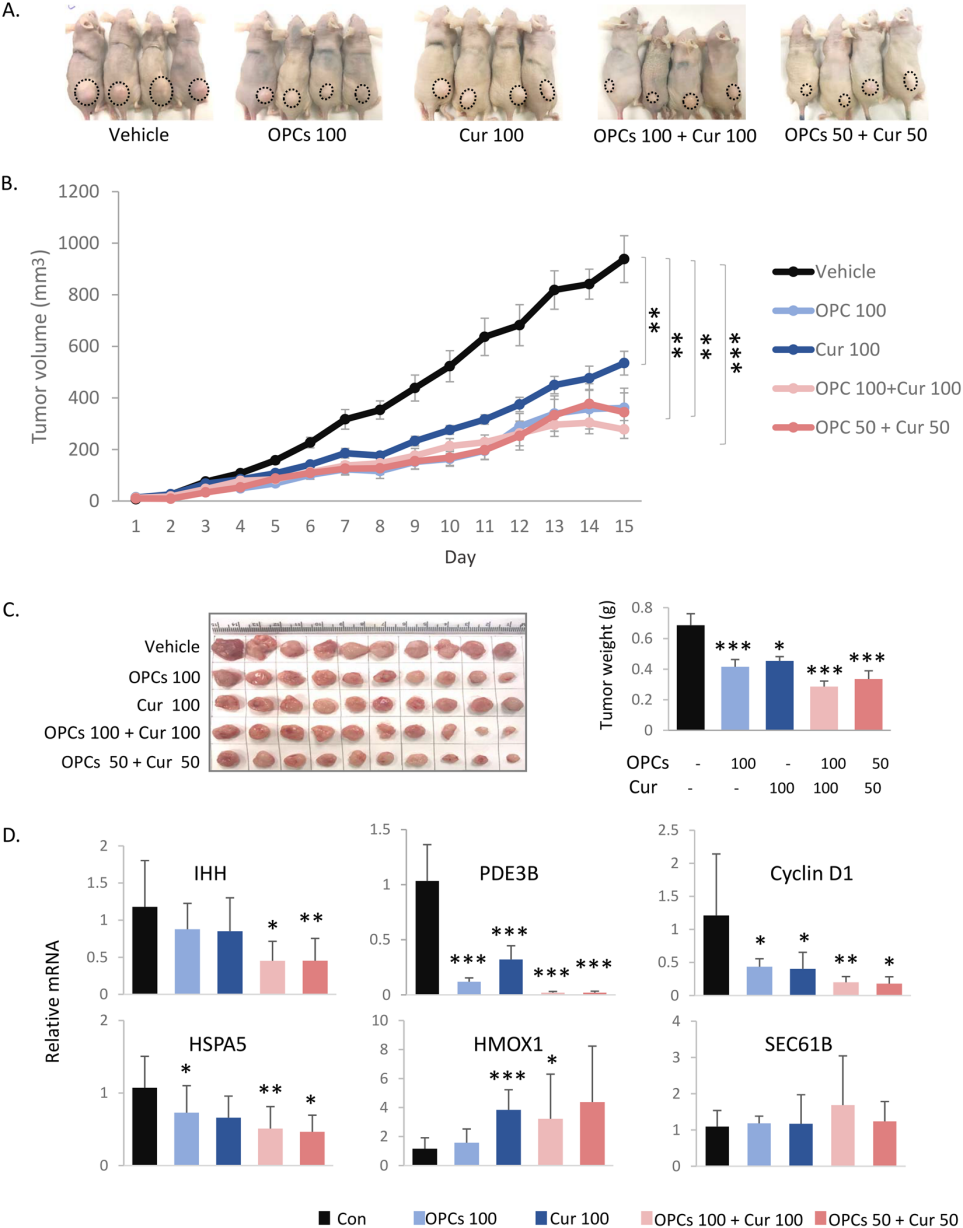
OPCs-curcumin combination effectively inhibits tumor growth in mice xenografts. (**A**) Representative images of mice with subcutaneous tumors 15 days after administering orally with curcumin, OPCs or in combination. (**B**) Progressive tumor volume in mice orally gavaged with OPCs and curcumin, individually and in combination. (**C**) Left: Xenograft tumors collected from sacrificed mice at the end of the 15-day treatments. Right: Quantification of tumor weights from different treatment groups. (**D**) qPCR analysis of mRNA levels of genes normalized to control group. mRNA levels of β-actin was used as the internal normalizing control. Indicated amounts of curcumin or OPCs is in mg/kg. *P < 0.05, **P < 0.01, ***P < 0.001 compared to control treatments.

To evaluate the effect of the combination of curcumin and OPCs *in vivo*, we followed the tumor growth in athymic mice with subcutaneous xenografts of HCT116 cells that were orally administered OPCs or curcumin alone, or in combination (Fig. 3A). There was no significant change in weight of the mice during the course of the treatment (Supplemental Fig. 4). While the tumors continued to grow in mice that were administered vehicle, tumor-growth in OPCs-administered mice (p = 0.005 for tumor volume, p = 0.005 for tumor weight) or curcumin-administered mice (p = 0.006 for tumor volume, p = 0.017 for tumor weight) was significantly attenuated (Fig. 3A,B). Interestingly, the combination of OPCs (100 mg/kg) and curcumin (100 mg/kg) was significantly more effective in decreasing tumor growth than the individual agents (p = 6 e-04 for tumor volume, p = 0.000214 for tumor weight; Fig. 3A–C). Interestingly, the combination of OPCs and curcumin at even lower doses of 50 mg/kg attenuated tumor growth as effectively as the higher doses of individual agents. Furthermore, in accordance with the results obtained in the cell lines, the combination of OPCs and curcumin decreased the expression of IHH, PDE3B, cyclin D1 and HSAP5, and increased HMOX1 and SEC61B more effectively than treatment with the singular compounds (Fig. 3D).

### OPCs inhibit tumor growth in patient-derived organoids

**Figure 4:**
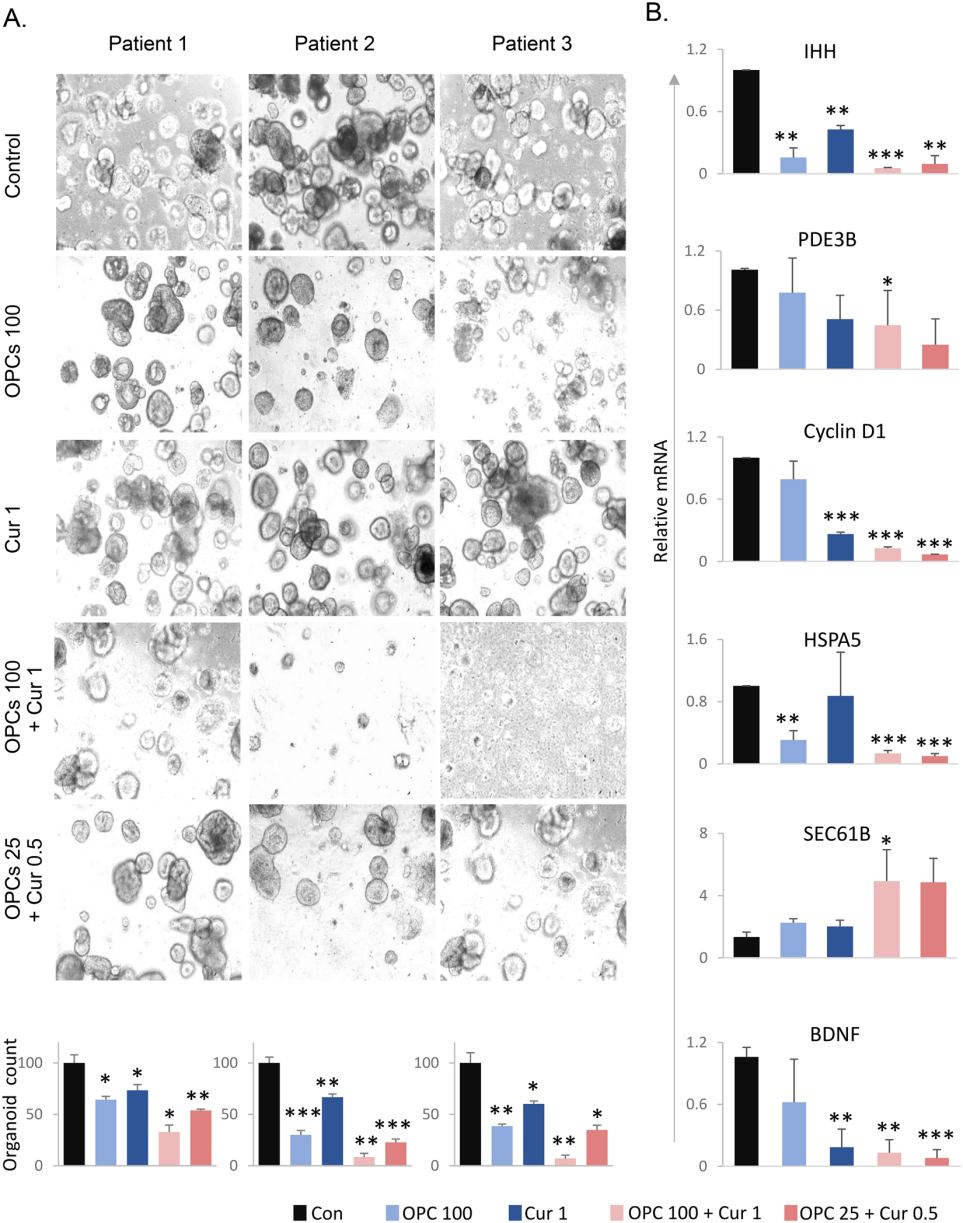
OPCs-curcumin combination effectively suppresses growth of organoids derived from human colorectal tumors. (**A**) Top: Images showing tumor organoid cultures derived from 3 different CRC patients, treated with OPCs and curcumin in combination or individually. Bottom: Bar graph showing decrease in spheroid count with treatments. (**B**) mRNA levels of genes in patient-derived organoids treated with OPCs and curcumin in combination or individually. mRNA levels of β-actin was used as the internal normalizing control. *P < 0.05, **P < 0.01, ***P < 0.001 compared to control treatments.

Tumor organoid model allows the maintenance and expansion of cells derived from patients in a 3D culture, and is physiologically superior to the conventional monolayer of cultured cells to study anti-cancer agents^44^. Therefore, we next used a tumor organoid model derived from CRC patients to further confirm our *in vitro* and *in vivo* observations. In line with our results in cell lines and mice xenografts, the OPCs-curcumin combination significantly decreased patient-derived tumor organoid formation and growth (Fig. 4A, top: representative images of organoids; bottom: organoid counts, p = 0.016 for patient 1, p = 0.003 for patient 2, p = 0.002 for patient 3). In line with our data from *in vitro* and *in vivo* experiments, the expression levels of IHH (p = 1.54 e-05), PDE3B (p = 0.04), cyclin D1 (p = 2.45 e-06), HSPA5 (p = 0.0003), SEC61B (p = 0.039) and BDNF (p = 0.0005) were significantly down-regulated by OPCs-curcumin combination in the patient-derived tumor organoids (Fig. 4B).

## Discussion

The cellular heterogeneity and the constant evolution of colorectal cancer complicates the design of effective treatment regimens. Drug combination therapies are becoming increasingly popular as they improve clinical outcomes^45^. When selecting drugs for combination therapies, it is beneficial to choose agents that work by targeting multitude of pathways with the potential likelihood of having additive or synergistic effects on the tumor and decreased chances of acquired resistance. In this study, by illuminating the cooperative molecular mechanisms of action of OPCs and curcumin, we contend that a therapeutic regimen combining OPCs and curcumin could effectively control colorectal cancer growth.

Several successful combinations of curcumin with other anti-cancer agents, including chemotherapeutic drugs and other natural compounds, have previously been explored and established. Curcumin considerably improves tumor-suppressive properties of chemotherapeutic agents, such as 5-fluorouracil, cisplatin and mitomycin^22–25^ and other phytochemicals, such as epigallocatechin-3-gallate from green tea^46^, boswellic acid from frankincense plant^47^ and quercetin from fruits and vegetables^48^. In this study, we not only showed an improvement in the anti-tumor properties of curcumin when combined with OPCs from grape seeds, but also delineated the molecular pathways through which curcumin and OPCs function cooperatively. Our data indicate that both OPCs and curcumin affect cell cycle and DNA replication, which are fundamental to cancer cell proliferation and progression. Cell cycle regulators, PCNA and cyclin D1, are hubs for interaction with other cancer-associated proteins^49^. Given the addiction of cancer cells to PCNA and cyclin D1, their targeted inhibition has been shown to successfully suppress cancer growth without affecting normal cells^50–52^. Likewise, targeting HSPA5 using small molecule drugs has proven to induce endoplasmic reticulum stress and apoptosis selectively in cancer cells, and not normal cells^53^. In our study, we observed that the expression of cyclin D1, PCNA and HSPA5 were attenuated by the OPCs and curcumin combination in CRC cell lines, mice xenograft tumors and patient-derived organoids. The inhibition of PCNA, cyclin D1 and HSPA5 expression by this combination is encouraging as a means to preferentially target cancer cells without affecting normal cells.

Our RNA-sequencing data revealed that while curcumin and OPCs commonly affect several genes, they also affect different genes within a pathway, thus effectively shutting off the entire signaling circuitry. By measuring mRNA levels using qRT-PCR, we confirmed that while certain genes, such as p21, were affected only by OPCs, other genes, such as SEC61B and PDE3B, were affected solely by curcumin. Intriguingly several key genes, such as G6PD and IHH, and pathways, such as protein export, notch and hedgehog signaling, were effectively regulated only by the combination of curcumin and OPCs, and not by the individual compounds. All these data demonstrate the potential of the OPCs-curcumin combination to block redundant cancer-associated pathways, which a main reason for the failure of conventional drugs^1,2^.

Several genes that were identified and validated in our study to be cooperatively modulated by OPCs and curcumin are also implicated in the development of chemo-resistance. SEC61B is essential for platinum drug accumulation, and its knockdown desensitizes cancer cells to oxaliplatin, carboplatin and cisplatin^38^, while G6PD and PDE3B enhance chemoresistance to oxaliplatin and cisplatin^39,54^. OPCs and curcumin together influenced several ABC transporters (Supplemental Fig. 3B) that impart resistance to multiple drugs by expelling drug molecules out of the cell. The intimation of the data that curcumin and OPCs could sensitize cells to chemotherapeutic agents remains to be studied systematically.

Collectively, in this study, we discovered that both curcumin and OPCs cooperatively affect several critical mechanisms of carcinogenesis such as DNA replication, cell cycle and mismatch repair. Apart from the commonly regulated pathways, our results highlighted the distinctive modes of actions of the two dietary agents; several pathways, such as Wnt-signaling and TGF-β signaling, were found to be uniquely modulated by OPCs, and not curcumin. Likewise, cellular mechanisms such as homologous recombination and nucleotide excision repair were modulated specifically by curcumin. The results from this study are important in the pre-clinical development of the OPCs-curcumin combination in colorectal cancer.

## Materials and Methods

### Cell culture and materials

Colorectal cancer cell lines, HCT116, SW480, SW620, HT29, RKO and LoVo were purchased from the American Type Culture Collection (Manassas, VA). All cell lines were tested and authenticated using a panel of genetic and epigenetic markers and tested for mycoplasma on a regular basis. The cells were grown in Dulbecco’s Medium Eagle’s medium (DMEM; Gibco, Carlsbad, CA), supplemented with 10% fetal bovine serum, 1% penicillin and streptomycin and maintained at 37 °C in a humidified incubator at 5% CO_2_.

Grape seed-OPCs (VX1 extract, EuroPharma, USA) and curcumin (BCM-95, Arjuna Natural Extracts, India) were dissolved in DMSO and diluted to appropriate experimental concentrations in culture medium.

### Cell viability and proliferation

Cells were plated in 96-well dishes at a density of 2000 cells/well in DMEM supplemented with 5% FBS and antibiotics, and allowed to attach overnight. Cell proliferation was measured in cells treated with a combination of OPCs (10, 100, 500, 1000 ng/ul) and curcumin (0.01, 0.05, 0.1, 0.5, 1, 2.5, 5) for 72 hours using WST-1 assay (Sigma-Aldrich) per manufacturer’s instructions. Each experiment was performed in triplicates.

### Cell cycle and apoptosis analysis

Cells plated in 24-well dishes were treated with OPCs or curcumin for 48 hours in triplicates. Cell cycle and apoptosis assays were performed using Muse Cell Cycle Assay kit (MCH100106, Millipore) and Muse Annexin V and Dead Cell Assay kit (MCH100105, Millipore) respectively, on Muse Cell Analyzer (Millipore) per manufacturer’s instructions.

### Patient-derived tumor organoids

Fresh tumor tissues were obtained from CRC patients enrolled at the Baylor University Medical Center, Dallas, and clinicopathologic characteristics of the patients from whom the CRC tissues were obtained is listed in Supplemental Table 1. The study was approved by the Institutional Review Board of Baylor Scott & White Research Institute, Dallas, TX. Written informed consent was obtained from all patients providing tissue specimens, and all experiments were performed in accordance with relevant guidelines and regulations proposed in the Declaration of Helsinki. CRC tumor organoids were cultured using a modified protocol described previously^55^. Briefly, following excision, tumors were maintained in a medium containing Advanced DMEM-F12 (Gibco) supplemented with 1% HEPES (Sigma-Aldrich), 1% L-glutamine (Gibco), 10% FBS (Gibco), 2% penicillin/streptomycin (Sigma-Aldrich) and 10 uM Y-27632 (R&D Systems). Tissues were minced and digested with collagenase solution (5 ml of above medium with 75 ul collagenase, 124 ug/ml dispase type II and 0.2% Primocen) for 30 min, then filtered through a 70 um filter (Corning). Cells were pelleted by centrifugation (200 g for 10 min), then suspended in Matrigel (BD Biosciences, Franklin Lake, NJ). Fifteen microliters of the cell-Matrigel suspension was placed in the center of 24-well plate and polymerized. A 1:1 mixture of L-WRN conditioned medium and DMEM/F12 medium (Gibco) supplemented with 20% FBS (Gibco), 2 mM L-glutamine (Gibco), 0.2% Primocen, 10 uM Y-27632 (R&D Systems), 10 uM SB431542 (R&D Systems) and 5% penicillin/streptomycin (Sigma-Aldrich) were added to the well and replaced every two days. For treatments, appropriate concentration of OPCs or curcumin or a combination of OPCs and curcumin were added to the culture medium and tumor organoids were allowed to grow for 1 week. The experiment was performed in triplicates.

The organoids were observed under a bright-field microscope. All 3D cell structures that were about 150–300 microns in diameter and that had a distinct epithelial cell layer were counted. Organoids in a certain plane of field were counted, while leaving out the ones that were out of focus.

### mRNA expression analysis

RNA from HCT116, SW480, SW620, RKO, LoVo and HT29 cells treated for 18 hours or tumor organoids treated for 7 days with DMSO (vehicle), OPCs (100 ng/ul), curcumin (1 ng/ul), OPCs(100 ng/ul) + curcumin(1 ng/ul) and OPCs(25 ng/ul) + curcumin(0.5 ng/ul), were isolated using mRNeasy kit (Qiagen). RNA from mice xenograft tumors collected in RNAlater solution (Qiagen) were extracted using mRNeasy Kit (Qiagen) following the manufacturer’s instructions. Extracted RNA was used as a template for cDNA synthesis using High Capacity cDNA Reverse Transcription Kit (ThermoFisher Scientific) according to manufacturer’s protocol. RT-qPCR was performed using SensiFAST SYBR mix (Bioline, London, UK) using the primer sequences listed in Supplemental Table 2. All RT-qPCR target genes were calculated using ΔΔCt method normalized to β-actin.

### Genomewide RNA Sequencing analysis

RNA from cell lines treated with DMSO or 100 ng/ul of OPCs or 1 ng/ul of curcumin or combination of OPCs (100 ng/ul) and curcumin (1 ng/ul) in duplicates were single-end sequenced. NGS library construction was performed using the TruSeq RNA Library Kit (Illumina) with up to 1 ug of total RNA input according to manufacturer’s protocol. The quality of individual libraries was assessed using the High Sensitivity DNA Kit (Agilent). Libraries were pooled together using a Pippin HT instrument (Sage Science). Efficiency of size selection was assessed using a High Sensitivity DNA Kit (Agilent). Pooled libraries were quantitated via qPCR using the KAPA Library Quantification Kit, Universal (KAPA Biosystems) prior to sequencing on an Illumina HighSeq 2500 with single-end 75 base read lengths. For the analysis of RNA-sequencing, Fastq files were trimmed using Flexbar to remove 3′ bases with quality scores lower than 30 before alignment, as described previously^56^. The trimmed reads were mapped to human genome version GRCH38 downloaded from GENCODE^57^ using HISAT2^58^ to generate alignment files in bam format. Samtools name-sorted bam files^59^ were processed using htseq-count to summarize gene level counts as described previously^60^. DESeq. 2 was used for differential gene expression analysis of RNA-sequencing read counts^61^. All sequencing data has been deposited to the GEO database (GSE109607).

Meta-analysis was performed using Stouffer’s p-value combination method^62^ to identify genes that are homogenously up or down regulated independently in OPCs-, curcumin- and OPCs + curcumin-treated cells. Fisher’s enrichment test on KEGG pathways was performed and the cellular pathways commonly regulated by both OPCs and curcumin with a p < 0.05 were identified. A similar meta-analysis approach was used to identify the cellular pathways that were uniquely regulated by OPCs or curcumin. Genes that were uniquely regulated by OPCs or curcumin or the combination of OPCs and curcumin were identified by Venn comparison. KEGG pathway analysis was done with these unique genes for OPCs, curcumin, OPCs-curcumin combination and pathways with p < 0.05 were plotted.

Additionally, KEGG pathway enrichment analysis was performed on genes whose fold change expression (with respect to untreated controls) in cells treated with OPCs-curcumin combination were higher than curcumin- or OPCs-only treated cells.

### Xenograft animal experiments

Seven week-old male athymic nude mice (Envigo, Houston, TX) were housed under controlled conditions of light and fed *ad libitum*. Approximately 1 × 10^6^ HCT116 cells were suspended in Matrigel matrix (BD Biosciences) and subcutaneously injected into mice using 27-gauge needle (n = 10 per group). Mice were randomly assigned to different treatment groups and orally gavaged with vehicle (glycerol:water, 1:1) or OPCs or curcumin (100 mg/kg body weight dissolved in vehicle) or OPCs-curcumin combination (100 mg/kg each and 50 mg/kg each) on alternative days for 15 days. Tumor size was measured each day by calipers. Tumor volume was calculated using the following formula: 1/2(length × width × width). The investigator was not blinded to the group allocation during the experiment and/or when assessing the outcome. The animal protocol was approved by the Institutional Animal Care and Use Committee, Baylor Scott & White Research Institute, Dallas, Texas and all experiments were conducted strictly in accordance to the National Institute of Health Guide for the Care and Use of Laboratory Animals (8th Edition Institute for Laboratory Animal Research).

### Calculation of cooperativity between agents

The following two methods described in the review article by Foucquier and Guedj^63^ were used to calculate cooperativity between curcumin and OPCs in cell proliferation assays:

- ‘Highest single agent’ (HSA) is the higher of the effects produced by each component in the combination at the same concentration as in the combination^64^. The agents are considered cooperative if the combined effect is in excess over the HSA. The Combination Index (CI) was calculated by dividing the maximum of the effect caused by the individual agents curcumin (E_cur_) or OPCs (E_OPCs_) by the effect by the combination of the agents (E_cur+OPCs_), i.e.,
  $${\rm{CI}}=[{\rm{\max }}({{\rm{E}}}_{{\rm{cur}}},{{\rm{E}}}_{{\rm{OPCs}}})]/{{\rm{E}}}_{{\rm{cur}}+{\rm{OPCs}}}$$
- ‘Bliss Independence’ model predicts the combined response for the individual agents. The agents are considered cooperative if the empirical effect of the combination is higher than the predicted effect^65,66^. To calculate the CI by this method, the product of the effects caused by the individual agents is subtracted from their sum, which is then divided by the effect caused by the combination of the two agents, i.e.,

$${\rm{CI}}=({{\rm{E}}}_{{\rm{cur}}}+{{\rm{E}}}_{{\rm{OPCs}}}-{{\rm{E}}}_{{\rm{cur}}}\cdot {{\rm{E}}}_{{\rm{OPCs}}})/{{\rm{E}}}_{{\rm{cur}}+{\rm{OPCs}}}$$

Two drugs with a CI value of <1 were considered to be cooperative.

### Calculation of dose reduction index (DRI)

DRI_50_ is a measure of the extent to which the concentration of OPCs or curcumin can be reduced at 50% inhibition of cell proliferation compared with the concentration of OPCs or curcumin individually^67^.

DRI_50_ for OPCs = IC50 for OPCs/Concentration of OPCs in combination for 50% inhibition.

DRI_50_ for curcumin = IC50 for curcumin/Concentration of curcumin in combination for 50% inhibition

In general, DRI > 1 is considered beneficial.

### Statistical analysis

All experiments were repeated three times. All data are expressed as mean ± SD with statistical significance indicated when *P* < 0.05. Statistical comparisons between control and treatment groups were determined using paired t-test.

## Electronic supplementary material

Supplementary Information
