# Supplementary material for: A combination of curcumin and oligomeric proanthocyanidins offer superior anti-tumorigenic properties in colorectal cancer
Source: Sci Rep. 2018 Sep 14;8:13869. doi: 10.1038/s41598-018-32267-8 (PMC6138725; doi:10.1038/s41598-018-32267-8)
Supplement: Supplementary file 1 — Supplementary Information [file 41598_2018_32267_MOESM1_ESM.pdf]

A combination of curcumin and oligomeric proanthocyanidins offer superior anti-tumorigenic properties in colorectal cancer

Preethi Ravindranathan[1], Divya Pasham[1], Uthra Balaji[2], Jacob Cardenas[2], Jinghua Gu[2], Shusuke Toden[1] and Ajay Goel[1]*

[1] Center for Gastrointestinal Research, Center for Translational Genomics and Oncology, Baylor Scott & White Research Institute and Charles A Sammons Cancer Center, Baylor University Medical Center, Dallas, Texas, USA

[2] Baylor Research Institute and Sammons Cancer Center, Baylor University Medical Center, Dallas, Texas, USA

Supplementary Figures and Tables

Suppl Figure 1. Calculation of Cooperativity: Highest single agent

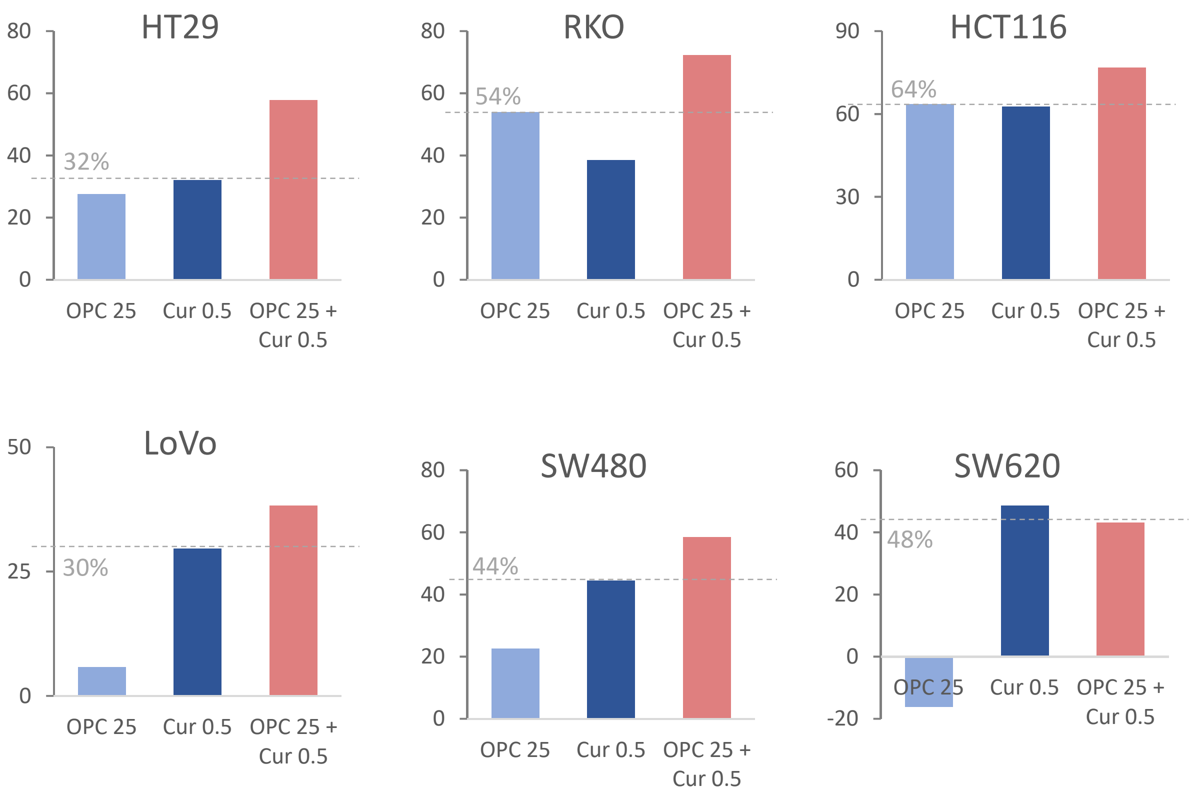

Cell line	CI index
HT29	0.55
LoVo	0.15
RKO	0.75
SW480	0.76
HCT116	0.83
SW620	1.12

Suppl Figure 2. Calculation of Cooperativity: Bliss Independence

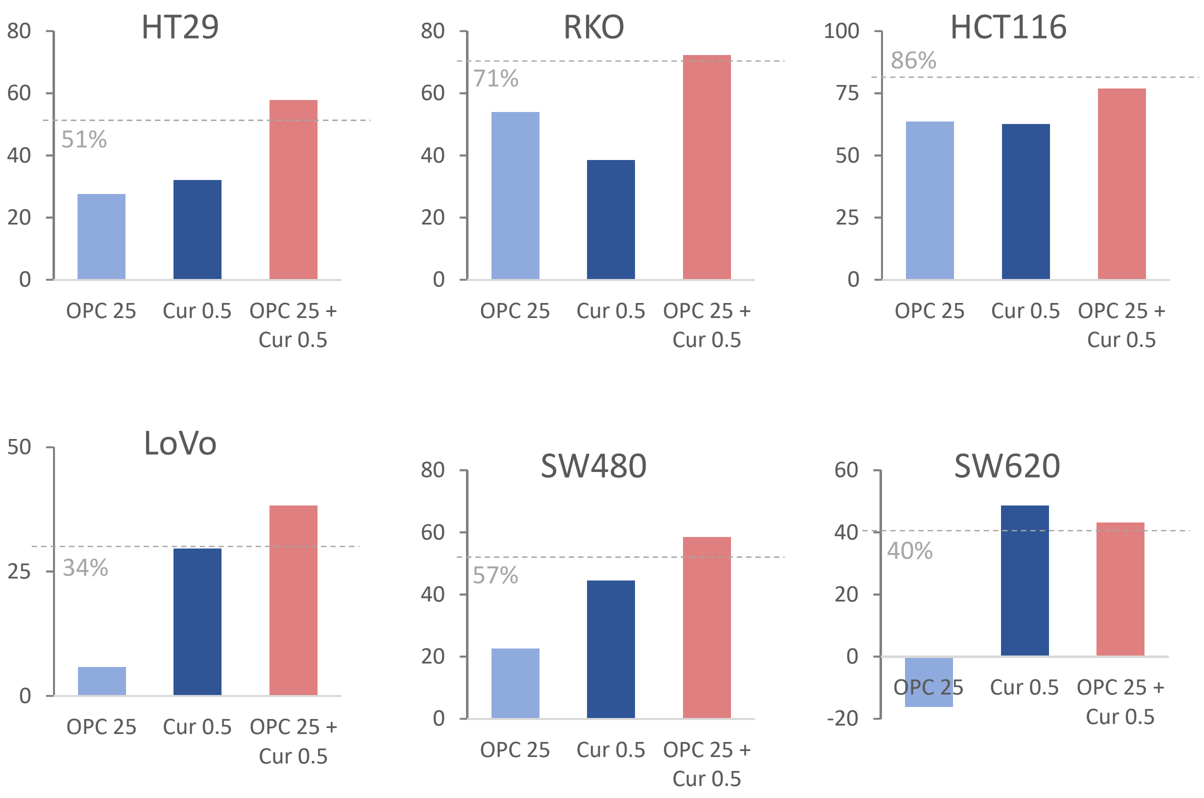

Cell line	CI index
HT29	0.88
LoVo	0.87
RKO	0.99
SW480	0.98
HCT116	1.12
SW620	0.93

Suppl Figure 3: Top pathways regulated uniquely by OPC, Curcumin and OPC-Cur combination

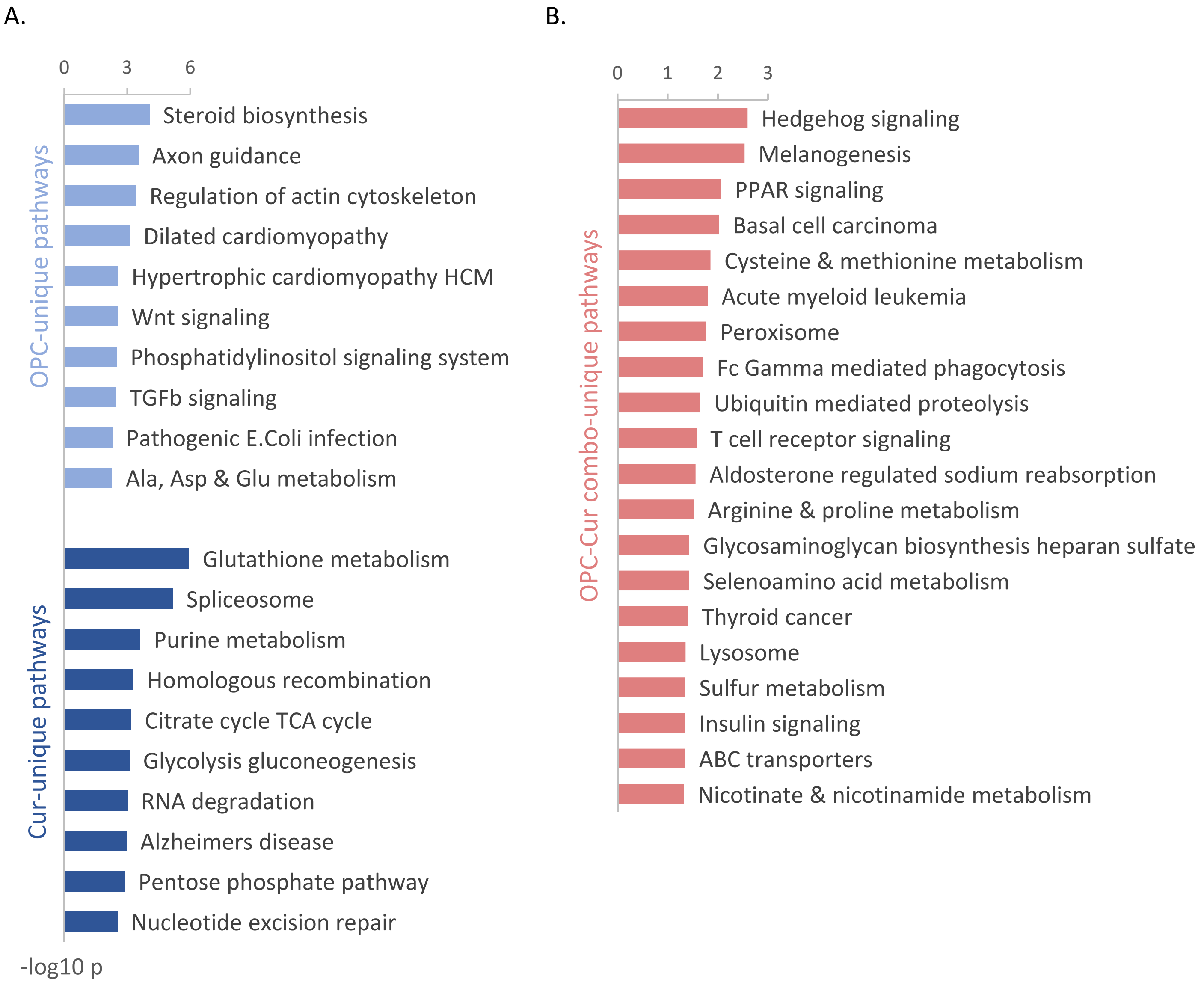

Suppl Figure 4: Animal weight during the course of treatment

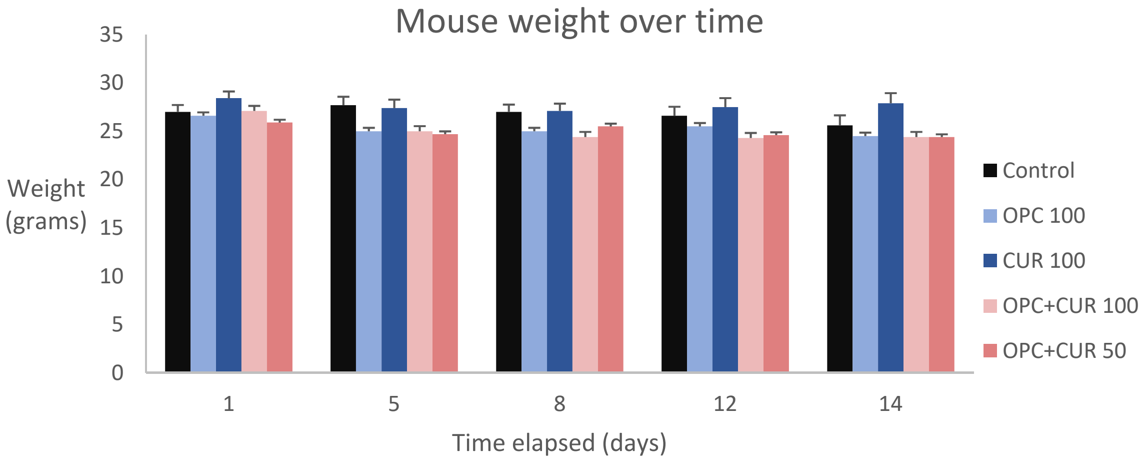

Suppl Table 1: Clinicopathologic characteristics of the 3 patients whose primary colorectal tumor tissues were used to develop organoid cultures

**Organoid**	**Patient Age**	**Gender**	**Tumor Location**	**Tumor Type**	**TNM stage**	**Pathology diff/ undiff**	**Local Invasion**	**Tumor Size(mm)**
#1	67	F	Cecum	Adenocarcinoma	pT4b N1b	moderate	present	40
#2	89	F	Cecum	Adenocarcinoma	pT3 N2b	moderate	present	56
#3	50	M	Sigmoid Colon	Adenocarcinoma	pT4b N0 M1a	moderate	present	64

Suppl Table 2: qPCR primers

Primer sequences	
PCNA-F	5'-CCATCCTCAAGAAGGTGTTGG-3'
PCNA-R	5'-GTGTCCCATATCCGCAATTTTAT-3'
CCND1-F	5'-CCATCCAGTGGAGGTTTGTC-3'
CCND1-R	5'-GTGGGACAGGTGGCCTTT-3'
E2F1-F	5'-AAGGGATTGGAGGCGTAGA-3'
E2F1-R	5'-CGCCAGTCAAACTGGTCTC-3'
p21-F	5'-AGGTGGACCTGGAGACTCTCAG-3'
p21-R	5'-TCCTCTTGGAGAAGATCAGCCG-3'
HSPA5-F	5'-TAGTGCAAGCTGAAGGCTGA-3'
HSPA5-R	5'-GATCTCGGCTCACTGCAAC-3'
SEC61B-F	5'-CTCATCTCCAATATGCCTGGT-3'
SEC61B-R	5'-CACTGCTTTGCTGGGAGAG-3'
G6PD-F	5'-TAGGCAGCCTCTCTGCTATAA-3'
G6PD-R	5'-GGGCTGTTTGCGGATTTAATG-3'
GCLC-F	5'-GACCCATGGAGGTGCAATTA-3'
GCLC-R	5'-CTGGTGAGCAGTACCACAAA-3'
HMOX1-F	5'-TCAGGCAGAGGGTGATAGAA -3'
HMOX1-R	5'-GCTCCTGCAACTCCTCAAA-3'
BDNF-F	5'-CCATGACCAGAAGGGAAACA-3'
BDNF-R	5'-CGGCAACAAACCACAACATTA-3'
IHH-F	5'-CTGAGCTGGGGGACACTG-3'
IHH-R	5'-GCCCAGTCAAGTCTCAATGG-3'
PDE3B-F	5'-GTGATGATGAAGACGGTGAAGA-3'
PDE3B-R	5'-TTCAGTGAGGTGGTGCATTAG-3'

Suppl Table 3: Dose-reduction indices

Cell line	DRI_{50} for OPCs	DRI_{50} for Curcumin
RKO	3.04	1.26
HCT116	3.47	0.28
LoVo	4.66	1.22
SW480	3.97	1.73
HT29	4.66	0.57
SW620	3.58	1.14

DRI_{50}: Drug Reduction Index at 50% efficacy for each agent
